# Mistletoe preparation (Viscum Fraxini-2) as palliative treatment for malignant pleural effusion: a feasibility study with comparison to bleomycin

**DOI:** 10.3332/ecancer.2014.424

**Published:** 2014-04-25

**Authors:** Rabab Gaafar, Abdel Rahman M Abdel Rahman, Fatma Aboulkasem, Ahmed El Bastawisy

**Affiliations:** 1 Medical Oncology, National Cancer Institute, Cairo University, Kasr El-Aini street, Fom El-khalig square, Cairo 11796, Egypt; 2 Surgical Oncology, National Cancer Institute, Cairo University, Kasr El-Aini street, Fom El-khalig square, Cairo 11796, Egypt

**Keywords:** mistletoe preparation, pleural effusion

## Abstract

**Background:**

Malignant pleural effusion is a common problem in patients with solid tumours. It has a significant impact on quality of life, and, hence, there is a substantial need to investigate new agents to treat it.

**Patients and methods:**

This is a prospective randomised controlled study, including patients with symptomatic recurrent malignant pleural effusion of different primaries. Patients were randomised into two groups: the first group received five ampoules of mistletoe preparation with defined lectin content (Viscum Fraxini-2, ATOS Pharma) diluted in 10 cc glucose 5% solution. Re-instillation was repeated every week until complete dryness of the pleural fluid was achieved (the maximum duration of the therapy was eight weeks). The second group received 60 units of bleomycin once intrapleurally.

**Aims:**

The primary aim of this paper was to evaluate the efficacy of mistletoe preparation as a palliative treatment for malignant pleural effusions in comparison with bleomycin. The secondary aim was to evaluate the tolerability of the mistletoe preparation.

**Results:**

A total of 23 patients were included and followed up during the study from December 2007 to January 2012: 13 patients received mistletoe preparation, and ten patients received bleomycin. Overall clinical response was reported in 61.5% of the mistletoe preparation arm versus 30% in bleomycin arm (*p* = 0.2138), 95% CI = (–0.1203, 0.6325). The toxicity of both arms was mild and manageable; the mistletoe preparation arm included fever, chills, headache, malaise, and, in two cases, allergic reaction, which was controlled by discontinuation of the drug and steroid injection.

**Conclusion:**

Mistletoe preparation is an efficient and well tolerated sclerosant agent which needs further investigation.

## Introduction

Pleural effusion is a frequent problem that has a great impact on the quality of life of cancer patients. Forty per cent of cases of pleural effusion are attributed to cancer with lung and breast primaries representing 75% of cases [1, 2]. Although control of the primary disease is the mainstay of treatment, most of these patients present with late stages of malignancy and mostly after exhaustion of multiple lines of systemic treatment, so local methods would be the only available method of treatment with tube thoracostomy and bedside pleurodesis [3]. Tetracycline (replaced by doxycycline), doxycycline, bleomycin, and talc are the most commonly used agents [4]. The preferred sclerosing agent is talc, which is more effective than bleomycin or tetracycline, according to European Society for Medical Oncology guidelines; however, the level of evidence is not very high (II, B), so bleomycin was used as a comparative in this paper, because it is the most frequent agent used in our institute. Viscum album with lectin, also known as European mistletoe, is a semiparasitic shrub that grows on other trees. Its extracts contain a number of biologically active compounds, mainly the mistletoe lectins (ML I, II, and III), viscotoxins, and other low molecular weight proteins, which exert immunomodulatry, cytotoxic, apoptotic, and antiangiogenic effects. It has been used in the treatment of cancer for the past 80 years [21].

## Aims

The primary aim of this paper was to evaluate the efficacy of mistletoe preparation as a palliative treatment for malignant pleural effusions in comparison with bleomycin. The secondary aim was to evaluate the tolerability of the mistletoe preparation.

## Patients and methods

This is a prospective randomised controlled study, including patients with symptomatic recurrent malignant pleural effusion of different primaries after discontinuation of active systemic treatment and referral to best supportive care facility. Patients were randomised into two groups. The first group received mistletoe preparation with defined lectin content (Viscum Fraxini-2). The second group received bleomycin once intrapleurally. The paper was conducted according to the Declaration of Helsinki and the guidelines for Good Clinical Practice. The local ethics committees approved the protocol, and informed consent was obtained from all patients before study entry.

### Inclusion criteria

The patient must have a histologically confirmed diagnosis of recurrent symptomatic malignant pleural effusion. The patient must be at least 18 years of age. The patient’s performance status must be (Eastern Cooperative Oncology Group Scale) ≤ 2. The patient must have adequate bone marrow function (white blood cell count ≥ 3.0 x109/L, absolute neutrophil count ≥ 1.5 x109/L, platelet count ≥ 100 x109/L, and haemoglobin level ≥ 9 g/L). The patient must have adequate liver function; serum bilirubin < 1.5 X upper limit of normal, alanine aminotransferase (ALT), and aspartate aminotransferase (AST) levels less than three times normal values. ALT and AST levels less than five times normal limits are allowed in patients with known liver metastases. The patient must have adequate kidney function, plasma creatinine level less than 1.5 times normal value. Patients should have compliance, mental state, and geographic proximity that allow adequate follow-up, and they have to provide written informed consent before any study-specific procedure.

### Exclusion criteria

The patient cannot be pregnant or breastfeeding. The study also excludes patients with a ‘currently active’ second malignancy; patients who are currently involved in another clinical trial; patients with previous unsuccessful pleurodesis, with pleural infection, or with chronic air leakage; and patients known or suspected to have hypersensitivity to mistletoe, uncorrectable bleeding tendency, or encysted pleural effusion.

## Pre-study evaluation

### Initial screening

For each eligible patient who consented to participate in the clinical study, the present and past history together with the clinical and radiological findings were collected.

### Basic investigation

Pathological confirmation of the diagnosis.

A pre-drainage base line posteroanterior and lateral chest radiograph (other imaging: CT scan or ultrasound of the chest as clinically indicated).

### Follow up

*Radiological:* posteroanterior and lateral chest radiograph was repeated weekly for six weeks, then after one month.

## Methodology

### In severe effusion (displacing mediastinal structures to contralateral side)

Eligible patients were admitted to the hospital, and a chest tube was inserted. Then, patients were randomised to intrapleural mistletoe preparation or bleomycin. The preparation consisted of 60 units of bleomycin, injected once, or five ampoules of mistletoe preparation (Viscum Fraxini-2) in 10 cc glucose 5% solution. Next, the tube was opened, and the drainage system placed under water seal for 24 h. For pleurodesis, when the 24-h tube debit is less than 200 ml, the sclerosing agent was injected into the pleural cavity through the chest tube, and the chest tube was then clamped for overnight then tube thoracostomy was removed. After tube withdrawal, the patient was discharged.

### In moderate and minimal effusion

Evacuation was done as much as possible using needle drainage. The sclerosing agent was injected into the pleural cavity through the needle, and then the needle was removed.

### The follow-up policy

The follow-up policy consisted of physical examination and chest radiography on a weekly basis after hospital discharge. In the mistletoe preparation (Viscum Fraxini-2) group: if there was still remaining pleural fluid it was evacuated, as much as possible, using a needle, then five ampoules Viscum Fraxini-2 (diluted in 10 cc glucose 5%) were re-injected intrapleurally every week until complete dryness of the pleural fluid occurred or the completion of six weeks. After six weeks if there was no response, patients were to be considered treatment failure and therapy was ended. If there was a minimal amount of fluid the patients continued the treatment for another two weeks (maximum duration of therapy eight weeks).

### Treatment response evaluation

Patients were assessed weekly for side effects, clinical examination, and the appropriate radiological investigations. Response evaluation was performed after six weeks from the start of therapy. Patients who showed increased pleural effusion (no subjective or objective response) after the first six weeks were to be considered treatment failure and to end therapy. These patients were then treated with other available tools at the discretion of the investigator. In cases of complete dryness (successful pleurodesis), patients were followed by chest x-ray after one month to assess if there was re-accumulation of the pleural fluid.

### Response criteria

Successful pleurodesis was defined as no recurrence of effusion on clinical and radiologic follow-up. The procedure was also considered successful in symptom-free patients with small residual effusion not requiring new thoracentesis.

### Toxicity

Toxicity evaluation was done according to the NCI Common Terminology Criteria for Adverse Events v4.0 (CT- CAE) [22].

### Statistical methods

SPSS package (version 17.0) was used for data analysis. Mean and standard deviation were reported to describe quantitative data. The Chi-square and Fischer exact tests were used to evaluate the differences in the distribution of the variables. P value of ≤0.05 was considered statistically significant.

## Results

A total of 23 patients presenting to the National Cancer Institute, Cairo University were included and followed up during the period from December 2007 to January 2012.

### Patient’s characteristics

Table 1 summarises patient’s characteristics with regard to number, age, sex, smoking history, histology, and degree of pleural effusion.

### Clinical response

There was numerically higher overall clinical response (61.5%) in the mistletoe preparation arm as compared with the bleomycin arm (30%), however, this was not statistically significant (*p* = 0.2138), 95% CI = (–0.1203, 0.6325). Two patients discontinued treatment upon their request after they experienced an allergic reaction during viscum injection and treatment was interrupted, so they were not included in the response assessment. (Table 2).

The number of viscum injections were as follows: one (three patients), two (five patients), three (one patient), four (one patient), six (three patients).

### Toxicity

Toxicity of both arms was mild and manageable, and included in the mistletoe preparation arm were: fever, chills, headache, malaise (all were grade 1), and in two cases allergic reaction, which did not necessitate hospitalisation and was controlled by discontinuation of the drug and steroid injection, however, those two patients decided to withdraw consent, and so were not included in the final analysis for response. Regarding the bleomycin group, patients suffered from mild fever and chest pain (all were grade 1). Other side effects or adverse reactions have not been identified.

## Discussion

Malignant pleural effusion is a common problem in patients with advanced malignancy of different diagnoses and reflects dismal prognosis. Many agents have been used for pleurodesis such as: tetracycline, doxycycline, bleomycin, and talc. Previous reports showed that chemical pleurodesis produces a complete response in 64% of patients with the success rate ranging from 0% with etoposide to 93% with talc with the following most reported adverse effects: pain (23%) and fever (19%) [7–12, 20].

Many of the adverse effects of the used agents are significant, for example, talc, which is one of the most effective sclerosing agents for treating malignant pleural effusions can cause severe pleuritis resulting in effective pleurodesis but on the other hand can worsen dyspnea and can also result in respiratory failure,fever, acute pneumonitis, granulomatous pneumonitis, and empyema [9–11].

We designated this paper to search for an alternative method of pleurodesis. Our study included patients with symptomatic recurrent malignant pleural effusion of different primaries. Patients were randomised into two groups: the first group received mistletoe preparation with defined lectin content (Viscum Fraxini-2). The second group received bleomycin intrapleurally. Our study showed a numerically higher overall clinical response (dry tap) (61.5%) in the mistletoe preparation arm as compared with the bleomycin arm (30%), however, this was not statistically significant (*p* = 0.2138). The toxicity of both arms was mild and manageable and included in the mistletoe preparation arm were: fever, chills, headache, malaise, and in two cases an allergic reaction, which was controlled by discontinuation of the drug and steroid injection.

Many previous preliminary studies using mistletoe preparations (Viscum Fraxini-2) for pleurodesis showed positive results with success rates lying between 70% and 96% with minimal side effects [13–19]. Our study adds to these positive results.

Chest tubes represent a physical and psychological burden on patients with advanced malignancy and pleural effusion, so in a case of mild and perhaps moderate pleural effusion we are in need of a method to perform effective pleurodesis with closed tapping without the need for the insertion of a chest tube, this approach was used in this paper and proved to be more convenient and helpful to the patients.

## Conclusions

We conclude that mistletoe preparation is an efficient and well-tolerated sclerosant agent for malignant pleural effusions of different primaries. In addition, patients with mild and moderate effusion can undergo closed tapping with mistletoe preparation without the need for a chest tube, this approach is more convenient for patients. Further studies including larger sample size are warranted.

## Figures and Tables

**Table 1.** Patient’s characteristics.

Character	Viscum number (%)	Bleomycin number (%)
Number	13	10
Age	32–68 years (median age = 50)	33–69 years (median age = 51)
Sex Male Female	7 (53.8) 6 (46.2)	6 (60) 4 (40)
Smoking Yes No	5 (38.4) 8 (61.6)	4 (40) 6 (60)
Histology Mesothelioma NSCLC(Adenocarcinoma) Small cell carcinoma Metastasis of unknown origin	8 (62) 3 (23) 1 (7.5) 1 (7.5)	3 (30) 5 (50) 0 (0) 2 (20)
Degree of effusion Moderate and minimal Severe	7 (53.8) 6 (46.2)	5 (50) 5 (50)

**Table 2.** Clinical response.

	Responding	Failed	Lost follow-up	Discontinuation	*P*-value
Viscum 13 (100%)	8 (61.5)	2 (15.4)	1 (7.7)	2 (15.4)	(*p* = 0.2138), 95% CI = (–0.1203, 0.6325)
Bleomycin 10 (100%)	3 (30)	4 (40)	3 (30)	–	
